# Transcriptome analysis of *Globodera pallida* from the susceptible host *Solanum tuberosum* or the resistant plant *Solanum sisymbriifolium*

**DOI:** 10.1038/s41598-019-49725-6

**Published:** 2019-09-13

**Authors:** Rinu Kooliyottil, Louise-Marie Dandurand, Joseph C. Kuhl, Allan Caplan, Fangming Xiao, Benjamin Mimee, Joël Lafond-Lapalme

**Affiliations:** 1 Department of Entomology, Plant Pathology and Nematology, University of Idaho, Moscow, ID 83844 USA; 2 Department of Plant Sciences, University of Idaho, Moscow, ID 83844 USA; 3 Agriculture and Agri-Food Canada, Horticulture Research and Development Centre, 430 boul. Gouin, St-Jean-sur-Richelieu, Quebec J3B 3E6 Canada; 4 McGill University Health Centre and Research Institute, 1001 Boulevard Décarie, Montréal, Quebec H4A 3J1 Canada

**Keywords:** Microbiology, Transcriptomics

## Abstract

A transcriptome analysis of *G*. *pallida* juveniles collected from *S*. *tuberosum* or *S*. *sisymbriifolium* 24 h post infestation was performed to provide insights into the parasitic process of this nematode. A total of 41 *G*. *pallida* genes were found to be significantly differentially expressed when parasitizing the two plant species. Among this set, 12 were overexpressed when *G*. *pallida* was parasitizing *S*. *tuberosum* and 29 were overexpressed when parasitizing *S*. *sisymbriifolium*. Out of the 12 genes, three code for secretory proteins; one is homologous to effector gene Rbp-4, the second is an uncharacterized protein with a signal peptide sequence, and the third is an ortholog of a *Globodera rostochiensis* effector belonging to the 1106 effector family. Other overexpressed genes from *G*. *pallida* when parasitizing *S*. *tuberosum* were either unknown, associated with a stress or defense response, or associated with sex differentiation. Effector genes namely Eng-1, Cathepsin S-like cysteine protease, cellulase, and two unknown genes with secretory characteristics were over expressed when *G*. *pallida* was parasitizing *S*. *sisymbriifolium* relative to expression from *S*. *tuberosum*. Our findings provide insight into gene regulation of *G*. *pallida* while infecting either the trap crop *S*. *sisymbriifolium* or the susceptible host, *S*. *tuberosum*.

## Introduction

Cyst nematodes have been identified as some of the greatest threat to agricultural crops worldwide^1^. The potato cyst nematodes (PCN) *Globodera pallida* Stone (1973) and *G*. *rostochiensis* Wollenweber, (1923) Skarbilovich, (1959) are found in potato production areas and are of regulatory significance throughout the world^1,2^. In the United States, *Globodera pallida* was first detected in 2006 in a potato processing facility in Idaho. Currently, there are no commercially acceptable potato varieties with *G*. *pallida* resistance suitable for production in the northwestern United States^3^.

*Globodera pallida* possesses highly sophisticated machinery to locate and parasitize its host plant^4–6^. Development and reproduction of potato cyst nematodes relies on the establishment and maintenance of a syncytium inside the host root. Once a feeding site is established, the nematode engages in a sustained biotrophic interaction with its host. Effectors, small proteins produced in the nematode’s esophageal glands, are delivered into plant cells through the stylet, and mediate this biotrophic interaction^4,7^. Effector proteins manipulate the host cell by modulating a variety of cellular processes such as suppression of host defense or stress responses and cause significant transcriptional re-programming in the host cell nucleus^8^. The early infection stages of the nematode life cycle are crucial in deciding the fate of the nematode. The ability of the nematode to overcome hostile conditions presented by the plant determines whether or not the nematode completes its life cycle. In a resistant host, the plant has acquired the capacity to recognize the parasite (often through the detection of specific effectors) to prevent the establishment of a feeding site. One response is the initiation of a hypersensitive response (HR) by the plant which triggers programmed cell death in infected cells^9–11^, but other processes may also be at work.

*Solanum sisymbriifolium*, a non-tuber bearing solanaceous plant, was identified as a promising trap crop for the control of *G*. *pallida*^12–14^. *Solanum sisymbriifolium* stimulates hatch of juveniles from the cysts, while preventing subsequent reproduction of *G*. *pallida*^15–17^. Microscopic observation revealed cell necrosis around the infective J2s in *S*. *sisymbriifolium* roots due to an HR as early as 2–4 days post infestation (dpi)^15^, and several dead nematodes were observed at this time point in *S*. *sisymbriifolium* roots. Due to unfavorable conditions in resistant plants, the nematode may experience stress and starvation. Nematodes are able to sense the physiological response of the plant and activate oxidative stress response pathways^18^. To evade the plant defense responses plant parasitic nematodes express an array of proteins such as antioxidants, catalases, peroxidases, heat shock proteins and body morphology and structural proteins^19,20^.

In this study, *G*. *pallida* juveniles that were parasitizing the roots of the susceptible host *S*. *tuberosum* and the resistant trap crop *S*. *sisymbriifolium* were extracted at 24 hours post infestation (hpi) and the transcriptome of these nematodes were analyzed using RNAseq methods. The transcriptome of *G*. *pallida* was studied at early stages of parasitism of because of the rapid defense response observed in *S*. *sisymbriifolium*^15^. This study has revealed several potentially important differences in the way the *Globodera* transcriptome changes according to the suitability of the plant as a host.

## Results

### Extraction of RNA from low numbers of nematodes

*Globodera pallida* juveniles were extracted from *S*. *tuberosum* or *S*. *sisymbriifolium* roots 24 h post infestation and a comparative transcriptome analysis was performed using RNAseq. Because an average of only 30 individual nematodes were isolated from *S*. *sisymbriifolium* roots 24 hours post infestation, the number of nematodes were normalized to 30 individuals from both the plant species for RNA isolation. Even though the samples were derived from just 30 nematodes collected from the roots of *S*. *sisymbriifolium* and *S*. *tuberosum*, sufficiently high-quality RNA (RNA quality number (RQN) > 8) was obtained for library preparation and sequencing.

### Sequencing quality analysis and mapping

Sequencing of three biological replicates of *G*. *pallida* extracted from *S*. *tuberosum* and *S*. *sisymbriifolium* generated a total of 402,702,714 paired-end reads (2 × 100 bp) at an average of 67,117,119 per sample. The average % mapping of sequence reads to the *G*. *pallida* reference genome was 19.21% or 18.06%, when the nematodes were parasitizing potato or *S*. *sisymbriifolium*, respectively. All the mapped RNA seq reads have been submitted to the National Center for Biotechnology Information (NCBI) with Sequence read archive (SRA) accession no. SRP159274.

### Differential gene expression of *G*. *pallida* parasitizing susceptible *S*. *tuberosum* or resistant *S*. *sisymbriifolium*

**Figure 1:**
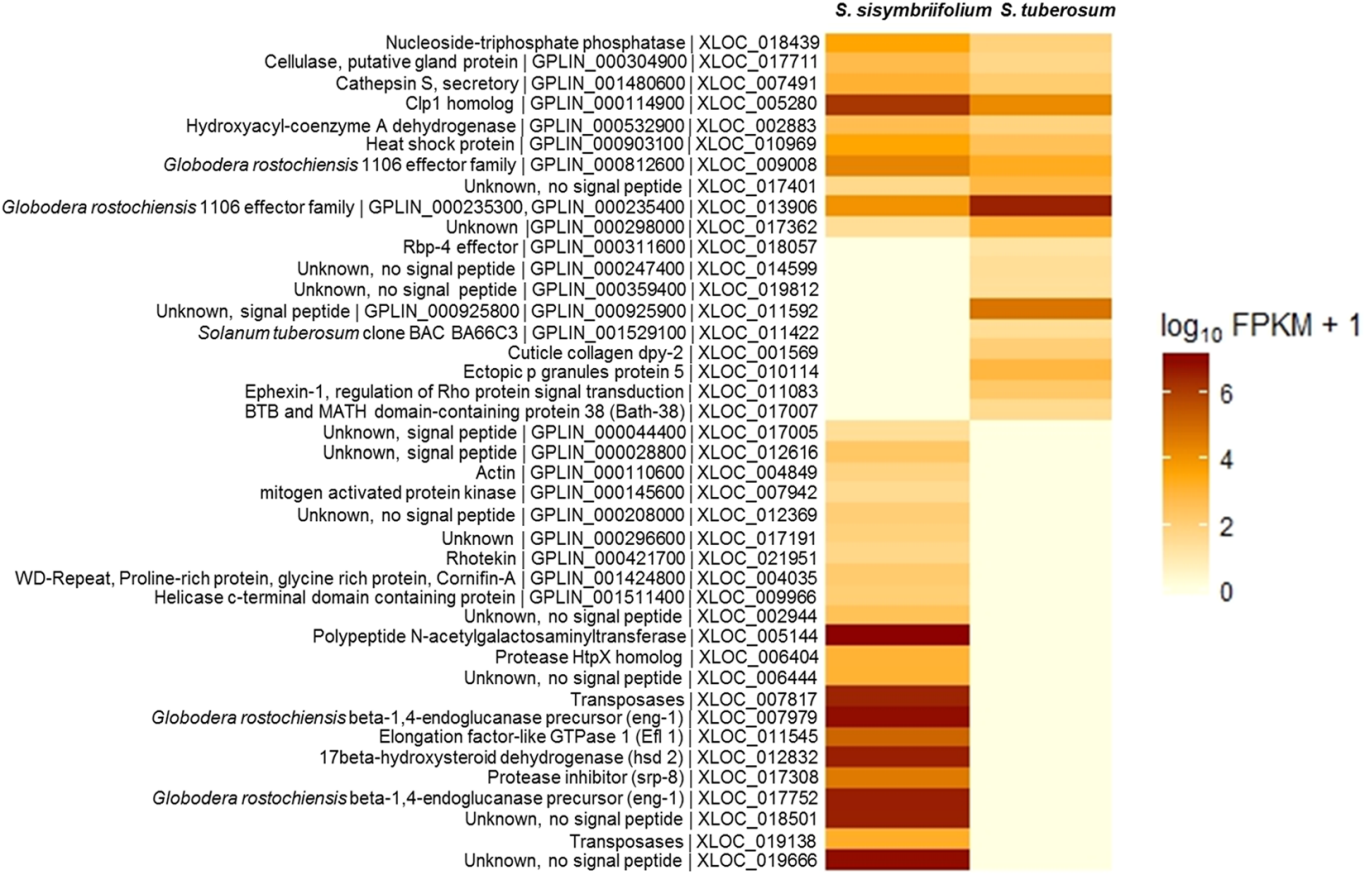
Heatmap showing the significant differentially expressed *Globodera pallida* genes when parasitizing susceptible *Solanum tuberosum* and resistant *Solanum sisymbriifolium* at 24 h post infestation. Each row represents a gene and column represents different plant species. Labels on the left show the transcript ID, followed by gene ID and closest gene name according to BLAST results. The color key is given on the right side of the heatmap.

For analysis of differential gene expression, only the reads that mapped to the reference *G*. *pallida* genome were considered. A total of 29,551 transcripts were identified across all the samples. Expression analyses identified 41 differentially expressed genes (DEGs) (P ≤ 0.01) (Fig. 1). Out of these 41 DEGs, 22 were expressed only when *G*. *pallida* was parasitizing *S*. *sisymbriifolium* and 9 were expressed only when parasitizing *S*. *tuberosum*. The remaining 10 genes among the 41 were expressed in both the cases with significant difference in expression values. Blast2GO analysis performed on these 41 DEGs returned 39% with annotations (see Supplementary Fig. S1). Gene ontology terms assigned to these transcripts are presented in Supplementary Fig. S2.

### Differential regulation of genes involved in nematode development, metabolism and sex differentiation

Functional analysis of the 41 DEGs revealed the overexpression of a protease coding gene Clp-1 (Ca2+ dependent cysteine protease calpain) when *G*. *pallida* was parasitizing *S*. *sisymbriifolium* (log2 fold change = 6.74). Other genes that were overexpressed when *G*. *pallida* was parasitizing *S*. *sisymbriifolium* were cathepsin S-like cysteine proteinase (log2 fold change = 3.38), an essential gene for invasion and reproduction, and the stress related gene hsp-20 (heat shock protein) (log2 fold change = 3.40). Genes encoding sex related proteins Hsd2 (hydroxysteroid 17-beta dehydrogenase 2) (no expression in *S*. *tuberosum*) and Srp-8, (signal recognition particle-8) (no expression in *S*. *tuberosum*) and genes encoding epidermal proteins cornifin-A (no expression in *S*. *tuberosum*) and cytoskeleton related actin (no expression in *S*. *tuberosum*) were also over expressed when parasitizing *S*. *sisymbriifolium*. Additionally, uncharacterized genes (without a signal peptide) namely, GPLIN_000296600, NAIXLOC_002944, NAIXLOC_006444, NAIXLOC_018501 and NAIXLOC_019666 were uniquely expressed when *G*. *pallida* was parasitizing *S*. *sisymbriifolium*. When *G*. *pallida* was parasitizing *S*. *tuberosum*, overexpression of genes with functions in autophagy (Epg-5, Ectopic p granules protein-5), nematode immunity (Bath-38 BTB and MATH domain containing protein), a guanine nucleotide exchange factor (GEF, Ephexin-1), and genes encoding cuticle collagen Dpy-2 were found. None of these genes were expressed when *G*. *pallida* was parasitizing *S*. *sisymbriifolium*.

### Differential regulation of effector genes

Among the 41 DEGs we found several characterized effectors as well as novel sequences with signal peptide which are putative effectors. Effector genes cellulase (log2 fold change = 3.55), cathepsin S-like cysteine protease (log2 fold change = 3.38), and *Globodera rostochiensis* 1106 effector ortholog (Gr1106, GPLIN_000812600) (log2 fold change = 4.26) were overexpressed when *G*. *pallida* was parasitizing *S*. *sisymbriifolium*. Other potential effector Eng-1 (Beta-1, 4-endoglucanase precursor) was only expressed when *G*. *pallida* was parasitizing *S*. *sisymbriifolium* but was not expressed when *G*. *pallida* was parasitizing *S*. *tuberosum*. Two other uncharacterized *G*. *pallida* gene sequences with signal peptide, GPLIN_000044400 and GPLIN_000028880 were also overexpressed when *G*. *pallida* was parasitizing *S*. *sisymbriifolium* but not when parasitizing *S*. *tuberosum*. Only, Rbp-4, and Gr1106 effectors (GPLIN_000235300) were overexpressed when *G*. *pallida* was parasitizing *S*. *tuberosum*. One *G*. *pallida* gene sequence of unknown function with a signal peptide, GPLIN_000925800, was expressed only when *G*. *pallida* was parasitizing *S*. *tuberosum* but not when parasitizing *S*. *sisymbriifolium*. The three above mentioned *G*. *pallida* genes (GPLIN_000044400, GPLIN_000028880 and GPLIN_000925800) with signal peptide did not show a dorsal gland promoter element motif (DOG Box) in their promoter region, which is a characteristic feature of dorsal gland cell effectors in *Globodera* species^7^. These genes need to be further studied for confirmation of their importance in parasitism.

### Confirmation of gene expression by qRT-PCR

**Figure 2:**
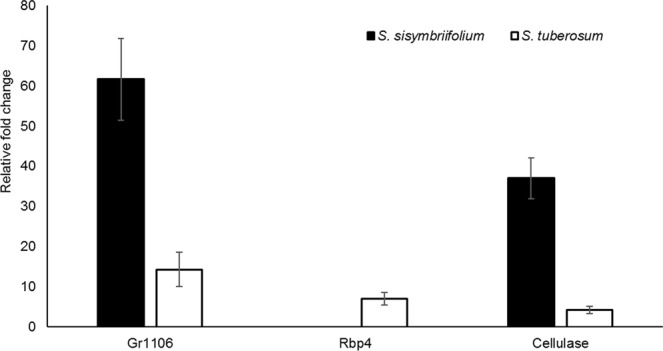
Quantitative PCR (qRT‐PCR) validation of RNA sequencing (RNAseq) data for the *Globodera pallida* genes 1106 orthologue, Rbp4 and cellulase during resistance and susceptible responses. The y axes represent the relative fold change (calculated using the ΔΔCt method) in gene expression. The data are representative of three independent biological replicates. Bars indicate standard errors.

To verify the RNA seq data, the expression profiles of the three *G*. *pallida* genes Rbp4 (GPLIN_000311600), Gr1106 (GPLIN_000812600), and cellulase (GPLIN_000304900) were determined by qRT‐PCR. A significant difference (P ≤ 0.05) in *G*. *pallida* gene expression was obtained when parasitizing *S*. *tuberosum* or *S*. *sisymbriifolium*. Similar to the results of the RNAseq differential expression analysis, qRT-PCR results indicated Gr1106 (47 fold higher, P = 0.02) and cellulase (32 fold higher, P = 0.04) in *G*. *pallida* was overexpressed when parasitizing S. *sisymbriifolium*, but expression of Rbp4 (7 fold higher, P = 0.02) when *G*. *pallida* was parasitizing *S*. *sisymbriifolium* was not detected (Fig. 2).

## Discussion

In this study the differential expression of transcripts involved in early stages of parasitism for *G*. *pallida* was determined by analyzing the transcriptome of *G*. *pallida* from either the susceptible host *S*. *tuberosum* or the resistant host *S*. *sisymbriifolium*. The functional analysis of the DEGs revealed the expression of genes important for parasitism such as proteases, cell wall modifying, stress related protein coding, and sex related protein coding. Several effector genes were also differentially expressed when *G*. *pallida* was parasitizing the susceptible host *S*. *tuberosum* or the resistant host *S*. *sisymbriifolium* at the early stages of parasitism. Of the 41 DEGs, 14 DEGs (either identified or uncharacterized) as illustrated in Fig. 1 (either known or unknown) have not been studied in relation to nematode parasitism.

Proteases play a variety of roles in host-parasite interactions, such as participation during invasion of host tissues, nutrition of the parasite, and escape from host defense responses^21^. A cysteine protease family gene Clp-1 and cathepsin S was highly expressed when *G*. *pallida* was parasitizing *S*. *sisymbriifolium*. The higher expression of Clp-1 and cathepsin S may be due to the defense response in the *S*. *sisymbriifolium* roots. Proteases have been identified as a primary target for the control of plant parasitic nematodes^22–25^.

This toxic environment induced by the host plant causes a stress response in the pathogen^18^. The stress response gene, Epg-5, was found to be over expressed when *G*. *pallida* was parasitizing *S*. *tuberosum*. A recent study reported the role of autophagy genes in the pine wood nematode *Bursaphelenchus xylophilus* as a response to stressful environmental conditions^26^. The gene, Epg-5, needs to be studied further to determine the expression over time when infecting *S*. *tuberosum*. Gene silencing studies will also provide information about the role of autophagy genes in the parasitism of *G*. *pallida*. One reason for the over expression may be to protect *G*. *pallida* at the early stages of parasitism. Earlier studies in other pathogens and insect pests show that autophagy has an important role in the growth, development, reproduction and pathogenicity for these organisms^27–30^. Epg-5, in human systems, encodes a large coiled coil domain-containing protein that functions in autophagy during starvation conditions^31^. Brennand *et al*. (2011) reviewed the strategy of human parasitic protists to starvation or stress conditions where autophagy plays a major role in adaptation to changes in the nutritional status of the cells^32^.

Heat shock proteins are stress tolerance proteins which are highly conserved in a wide range of organism and are reported to be essential candidate genes which can be targeted for plant mediated resistance development^33,34^. Over expression of the heat shock protein gene hsp-20 was also observed when *G*. *pallida* was parasitizing *S*. *sisymbriifolium*. Hsp-20 is in the small heat shock protein family (sHSPs), which function as molecular chaperones. Under stress conditions, sHSPs bind to denatured proteins and maintain them in a folded state which prevents the irreversible aggregation of these proteins. When stressful conditions such as starvation, high temperature etc. are removed, sHSPs are released and re-naturation takes place^35,36^. This may be an attempted adaptive mechanism for *G*. *pallida* to overcome the stress encountered when invading *S*. *sisymbriifolium* roots. Metabolic pathway analysis revealed that heat shock protein (hsp) hsp-20 was classified under both stress related and sex related protein categories. However, detailed research would need to be conducted to determine the relationship between hsp and sex determination for plant parasitic nematodes. Two other genes encoding sex related proteins namely, Hsd-2 and Srp-8 were also overexpressed when *G*. *pallida* was isolated from *S*. *sisymbriifolium*. Although this may not be the case for *G*. *pallida* while infecting *S*. *sisymbriifolium*, in some resistant plants delayed HR known as male-based resistance is evident, and results in sexual differentiation to males^37^. The relationship of stress and sex differentiation is poorly understood and needs to be further studied.

Overexpression of *G*. *pallida* genes encoding epidermal proteins (cornifin-A), and cystoskeleton (actin) while infecting *S*. *sisymbriifolium* was observed, possibly due to changes in the cytoskeleton and body wall muscles under stress conditions. In a resistant plant such as *S*. *sisymbriifolium* a feeding cell is not formed which leads to starvation‐induced stress in nematodes and, subsequently, cessation of their development^19^.

Effector genes modulate host metabolism for a successful infection and life cycle completion. Effector molecules which protect nematodes from plant defenses called immune-modulators play decisive roles in a successful parasitism^4,5,38,39^. Plants have evolved defense proteins that can recognize pathogen effectors, and induce ETI^40,41^, often involving a type of cell death known as the hypersensitive response (HR). An HR to *G*. *pallida* was observed in *S*. *sisymbriifolium*^15^ (Supplementary Fig. S3). In the present study, genes with secretory characteristics were identified as Rbp-4, *Globodera rostochiensis* 1106 effector (Gr1106), and one uncharacterized gene with a signal peptide. These genes were found to be highly expressed when *G*. *pallida* was parasitizing *S*. *tuberosum*. Another member of the Gr1106 effector family GPLIN_000812600 was found to be highly expressed when *G*. *pallida* was isolated from *S*. *sisymbriifolium*. The *G*. *rostochiensis* effectors 1106 (Gr1106 effectors) suppress effector-triggered immunity (ETI) for development of the nematode in susceptible plants^42^. Although detailed studies are not available on the Rbp-4 gene, a closely related (protein sequence similarity is presented in Supplementary Fig. S4) *G*. *pallida* gene, Rbp-1 (*Gp*-Rbp-1), has been found to provoke an ETI response from potatoes when it interacts with Gpa2, a resistance protein containing coiled- coil, nucleotide-binding, and leucine-rich-repeat domains^9,43^.

During nematode invasion and migration, cell wall‐degrading or modifying enzymes including endoglucanases (glycosylhydrolase (GH) family) are expressed^39^. Higher expression of cell wall-modifying enzymes coding effector genes beta-1,4-endoglucanase (Eng-1 and cellulase) and hydroxyacyl-coenzyme A dehydrogenase was observed when *G*. *pallida* was parasitizing *S*. *sisymbriifolium* compared to when *G*. *pallida* was parasitizing *S*. *tuberosum*. Eng-1 was not expressed when *G*. *pallida* was parasitizing *S*. *tuberosum*. The over expression of GH family enzymes in *G*. *pallida* when infecting a resistant plant species compared to their expression in susceptible plant species could be attributed towards the structural complexity of plant cell wall, however a detailed study is required to better understand this phenomenon. Shukla *et al*. (2018) also reported the over expression of GH family genes in resistance response^19^.

In conclusion, this study provides insight into the molecular mechanisms of a susceptible and resistant interactions at early stages of *G*. *pallida* parasitism. Our results indicate that, at an early stage of infection, over expression of several effector genes, including cell wall modifying enzyme coding genes, may play a role to overcome plant defenses from *S*. *sisymbriifolium* in *G*. *pallida*. We have also observed over expression of several stress related genes when *G*. *pallida* was isolated from *S*. *sisymbriifolium*. The role of these genes in parasitism is poorly understood and need further investigation. Several novel and known nematode genes were identified, which are differentially expressed when *G*. *pallida* was infecting a resistant or susceptible plant species, which will help to advance research in plant-nematode interactions. Genetic engineering experiments aimed at creating nematode resistant potatoes may benefit by targeting DEGs produced during the earliest stages of parasitism.

## Materials and Methods

### Rearing of *G*. *pallida*

*Globodera pallida* cysts collected from infested fields in Shelly, ID, USA were reared on a susceptible potato cultivar ‘Desiree’ in clay pots filled with sterilized sandy loam soil and sand (2:1) under greenhouse conditions of 18 °C ± 2 °C (day time), 14 ± 2 °C (night time), and 16:8-h light: dark period^12,15,44^. After 16 weeks, cysts for experimental use were recovered by extraction from soil using the Fenwick Can method^45^. Prior to experimental use, all cysts were incubated at 4 °C for a minimum of 16 weeks. For the current experiment the cysts were put inside sterile nylon 250 μm mesh bags (McMaster Carr, Elmhurst, IL). The nylon mesh was sealed along the edges with a hand sealer (Sealer 8″ F-200, Sealer sales Inc., Northridge, CA), surface sterilized in a solution of 0.5% NaOCl for 5 min and rinsed thoroughly with sterile distilled water. The cyst bags were then amended with potato cultivar ‘Desiree’ root diffusate (collected from 4 weeks grown plants) containing gentamicin (1.5 mg/ml) and nystatin (0.05 mg/ml) and incubated at 20 °C in a sterile 6-well plate. After 2 weeks the hatched juveniles (J2s) were collected and surface sterilized in 100 μg/ml of ampicillin and streptomycin (w/v) and in 0.125% w/v benzethonium chloride^15,46^.

### Plant materials

*Solanum tuberosum* L. cv. ‘Desiree’ were propagated in standard tissue culture media^47^. After 4 weeks the tissue culture plants were transplanted into root trainers (Haxnicks™, TDI Brands, USA) and kept under greenhouse conditions 18 °C ± 2 °C (day time), 14 ± 2 °C (night time), 16:8-h light: dark period. *Solanum sisymbriifolium* seeds (Accession PI 381291), were obtained from Chuck Brown, USDA‐ARS, Prosser, WA, USA and were grown in plastic seed trays for 4 weeks before being transplanted into root trainers and kept under greenhouse conditions, as above. Both the plant species were grown in root trainers for 4 weeks prior to inoculation.

### Infecting plants with *G*. *pallida*

Four weeks post planting, root trainers were opened and single root from each plant was inserted into a glass tube (10 cm L × a.5 cm W, Supplementary Fig. S5). The tube was then filled with sterilized sandy loam soil and sand (2:1) mix. Surface sterilized *G*. *pallida* J2s suspension (1500 J2s/ml) in 0.2% agarose was inoculated onto the single root inserted in to the glass tube. There were 3 replicates for each plant species.

### Extraction of infected *G*. *pallida* from the roots of *S*. *tuberosum* and *S*. *sisymbriifolium*

The infected roots were removed from the tube, and gently washed 24 h post- infestation. The washed roots were cut into 1 mm length and blended for 20 seconds in a blender (WARING Commercial®, WARING Products Division, CT, USA) on a low speed setting with ice cold water. The suspension was passed through three sieves stacked together (no. 60 on top, 200 in the middle and 500 at the bottom). The nematodes were collected from the no. 500 sieve and transferred to a Petri dish (modified from Coolen and D’Herde 1972^48^). Under a microscope (Leica, M 80, Germany) the nematodes were pipetted individually using a Pasteur pipette into an Eppendorf tube (kept on ice). Approximately 30 nematodes were isolated from *S*. *sisymbriifolium* root samples, therefore, only 30 nematodes from each plant species were used for RNA extraction. The extra water was pipetted out from each tube and the nematodes were immediately frozen in liquid nitrogen and stored at −80 °C.

### RNA isolation, sequencing and differential expression analysis

A previously published RNA extraction protocol was modified by supplementing frozen nematode samples with 200 µl Proteinase K solution (100 µg/ml Proteinase K in 1% sodium dodecyl sulfate, 50 mM Tris, pH 7.5, and 10 mM Ethylenediaminetetraacetic acid (EDTA) - pH 8, 5% β-mercaptoethanol)^49^. The suspension was then incubated at 50 °C for 15 minutes, and then mixed with 500 µl RNAzol (RNAzol® RT, Sigma-Aldrich, USA), and incubated at room temperature for 15 min. The suspension was next centrifuged at 13,000 g for 12 min and the supernatant was transferred to a new tube. The volume of supernatant was measured and precipitated by mixing freshly made 70% ethanol to bring the final solution to 40% ethanol. The tubes were incubated at room temperature for 45 min and centrifuged at 21,000 g for 8 min^49^. After removal of the supernatant, the pelleted RNA was purified by adding 400 µl magnetic bead buffer provided in the Agencourt RNAdvance Tissue total RNA extraction kit (Beckman Coulter, USA). RNA was further purified as per manufacturer protocol (Agencourt RNAdvance Tissue total RNA extraction kit, Beckman Coulter, USA). The final elution was done in 20 µl RNAse free water. RNA quality and quantity were estimated on a FRAGMENT ANALYZER™ (Advanced Analytical Technologies Inc., USA) and Qubit™ 3 fluorometer (Life Technologies, USA). Only samples with good RNA quality (RQN > 8) were kept for further analyses.

For each sample an average of 25 ng total RNA was used for library preparation using TruSeq® Stranded mRNA Library Prep Kit for NeoPrep™ (Illumina, San Diego, USA). The libraries were sequenced on Illunima HiSeq 4000 (100 bp paired end (PE)) at the QB3 Vincent J. Coates Genomics Sequencing Laboratory, University of California, Berkely, CA, USA. The 100 bp PE reads were assessed for quality using FastQC program^50^. Trimmomatic was used for trimming the adapters and primers from the reads^51^. The quality trimmed reads were processed through the Tuxedo suite^52^ for differential gene expression analysis by aligning the reads onto the *G*. *pallida* genome, G.pal.v1^53^ downloaded from Welcome Trust Sanger Institute data base using tophat v2.0.14^54^ on default parameter settings. The aligned reads were further processed through cufflinks, cuffcompare, cuffmerge and cuffdiff of Tuxedo suite. The cuffdiff output data was visualized on an R program cummeRbund^55^. Pairwise similarities between conditions and replicates were visualized using the csDistHeat function on the cummeRbund program (Supplementary Fig. S6). A confidence level of 99% (P = 0.01) was set to evaluate the significant difference in *G*. *pallida* gene expression extracted from two different plant species 24 hours-post-infestation.

### Functional characterization of transcripts

To investigate functions, the transcripts were processed using Blast2GO PRO (version 4.1.9) software^56^. The data was processed through Cloud BLAST (bastx-fast, e-Value = 1.0E-5) of Blast2GO. In addition, significant differentially expressed gene (DEG) sequences were individually extracted and blasted (blastn) to the NCBI database. Those transcripts without a gene annotation name from the differential expression data set were blasted to Wormbase (http://parasite.wormbase.org/Multi/Tools/Blast) to compare with *G*. *pallida* genome data. Transcripts that did not overlap with annotated genes (during the cufflinks step) are given an ID, that is XLOC, instead of GPLIN. Transcripts were also translated using ExPASy (http://web.expasy.org/translate/) and blasted to ExPASy BLAST (http://web.expasy.org/blast/) to confirm their identity. To predict possible effector genes among the differential expression data, the protein sequences were assessed for signal peptides using SignalP 4.1^57^.

### Gene expression analysis using quantitative PCR (qRT-PCR)

To validate DEGs on qRT-PCR, three potential candidate effector genes were selected namely, Rbp4, 1106 ortholog and cellulase from the DEGs list. The *G*. *pallida* infected roots of *S*. *tuberosum* and *S*. *sisymbriifolium* were extracted 24 h post infestation, and RNA was isolated as per the methods described above. An average of 25 ng total RNA was used for cDNA synthesis using SUPERSCRIPT^®^ II reverse transcriptase (Invitrogen) on a Thermal Cycler C1000™, (BIO-RAD, USA). The qRT-PCR (ViiA™ 7 Real-Time PCR System, Applied Biosystems, USA) reaction was performed in 20 µL total volume containing 10 µL master mix (FAST SYBR™ Green Master Mix, Thermo Fisher Scientific Inc, MA, USA), 1 µL template (10 ng), 200 nM (primer efficiency = 104.11, R^2^ = 0.98) each of forward and reverse primers of elongation factor (reference gene), 150 nM (primer efficiency = 99.65, R^2^ = 0.98) each of forward and reverse primers of RBP4, 200 nM (primer efficiency = 100.51, R^2^ = 0.98) each of forward and reverse primers of 1106 ortholog, 200 nM (primer efficiency = 101.88, R^2^ = 0.97) each of forward and reverse primers of cellulase, and nuclease free water was added. The cycling conditions included 95 °C for 2 min, 40 cycles of 95 °C for 15 sec, and an annealing step for 30 sec at 55 °C. The relative expression level was calculated in comparison to elongation factor by means of 2^(control sample Ct of target−infected sample Ct of target)^ ÷ 2^(control sample Ct of reference−infected sample Ct of reference)^^58^. The results presented are the average of three biological replicates. T-test was performed to determine significant differences between the relative expression of *G*. *pallida* genes when isolated from *S*. *tuberosum* and *S*. *sisymbriifolium*.

## Supplementary information

Supplementary Information

## Data Availability

The aligned RNAseq reads are available at National Center for Biotechnology Information (NCBI) with Sequence read archive (SRA) accession no. SRP159274.
