# Supplementary material for: Transcriptome analysis of Globodera pallida from the susceptible host Solanum tuberosum or the resistant plant Solanum sisymbriifolium
Source: Sci Rep. 2019 Sep 13;9:13256. doi: 10.1038/s41598-019-49725-6 (PMC6744408; doi:10.1038/s41598-019-49725-6)
Supplement: Supplementary file 1 — Supplementary Information [file 41598_2019_49725_MOESM1_ESM.pdf]

Transcriptome analysis of *Globodera pallida* from the susceptible host *Solanum tuberosum* or the resistant plant *Solanum sisymbriifolium*

Rinu Kooliyottil, Louise-Marie Dandurand, Joseph C Kuhl, Allan Caplan, Fangming Xiao, Benjamin Mimee, and Joël Lafond-Lapalme

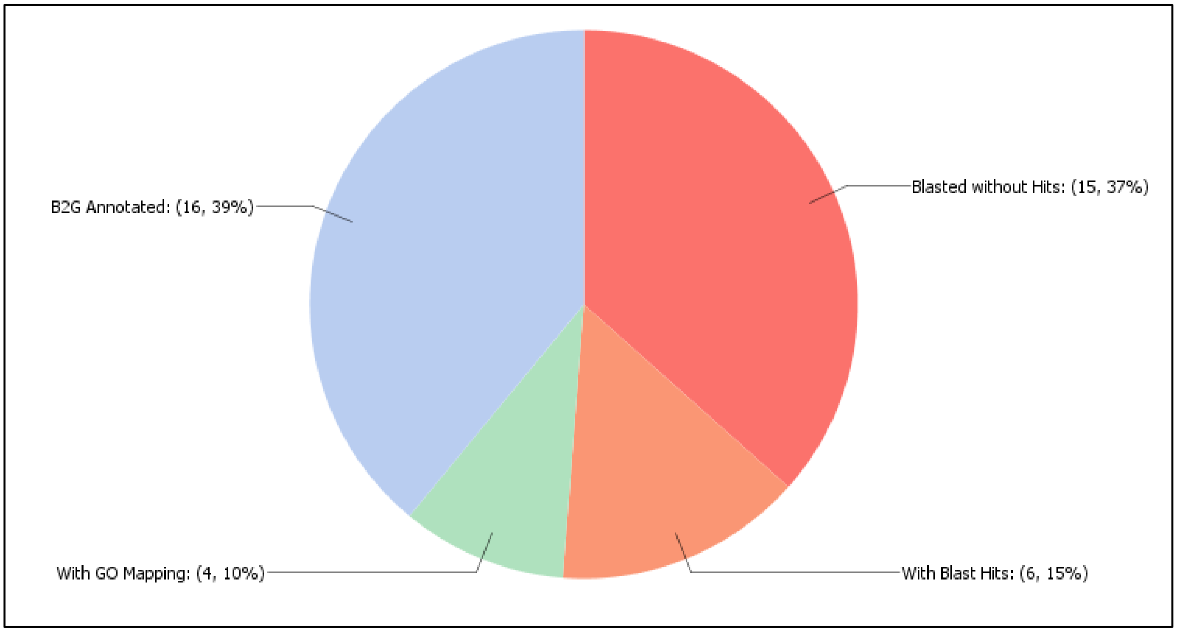

Fig. S1. BLAST statistics of the 41 differentially expressed genes of *Globodera pallida* isolated from *Solanum tuberosum* and *Solanum sisymbriifolium* 24 h post infestation.

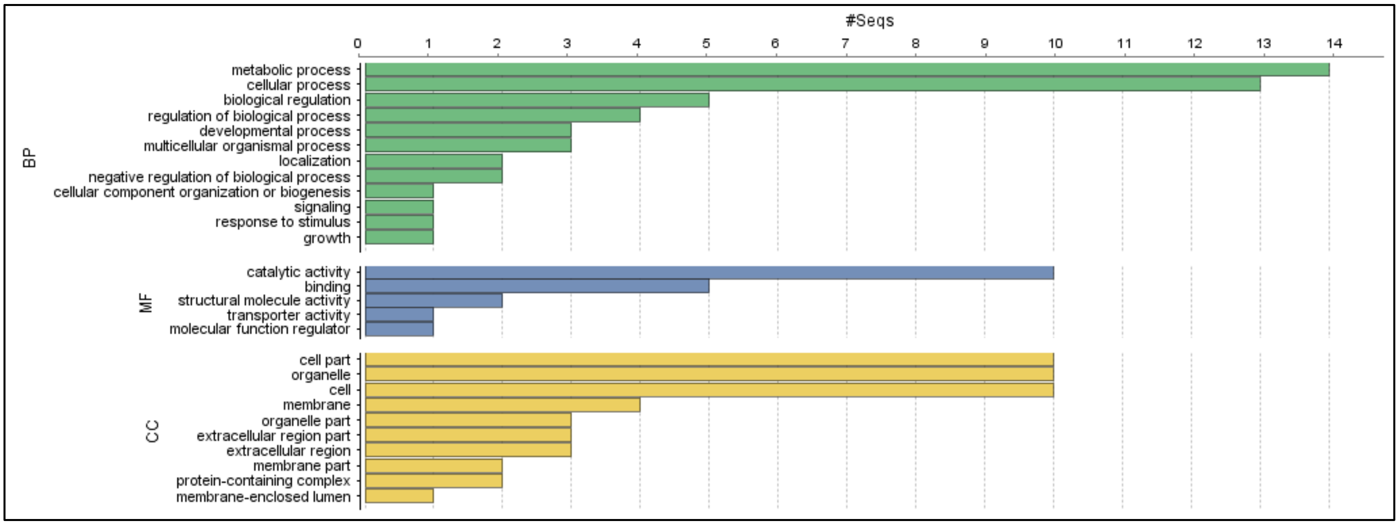

Fig. S2. Distribution of GO annotation terms for biological process (BP), molecular function (MF) and cell compartment (CC) in the differentially expressed genes of *Globodera pallida* isolated from *Solanum tuberosum* and *Solanum sisymbriifolium* 24 h post infestation.

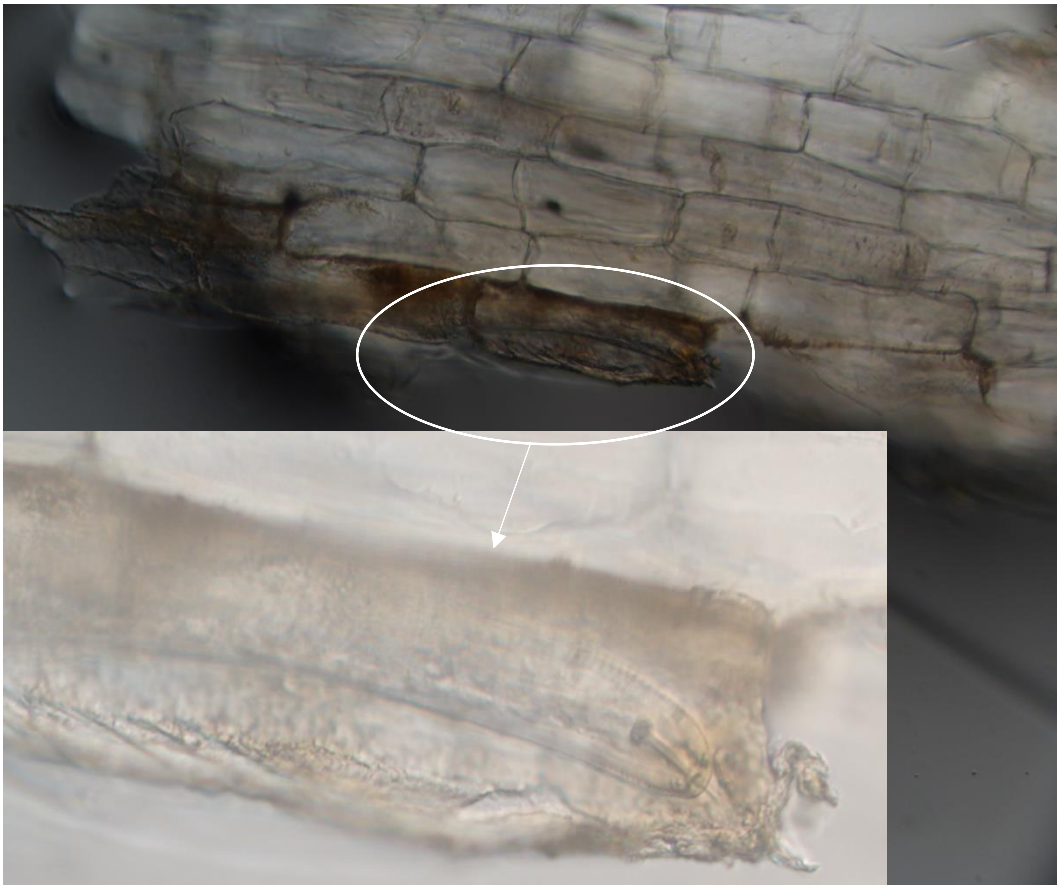

Fig. S3. *Globodera pallida* invading a root cell of *Solanum sisymbriifolium*.

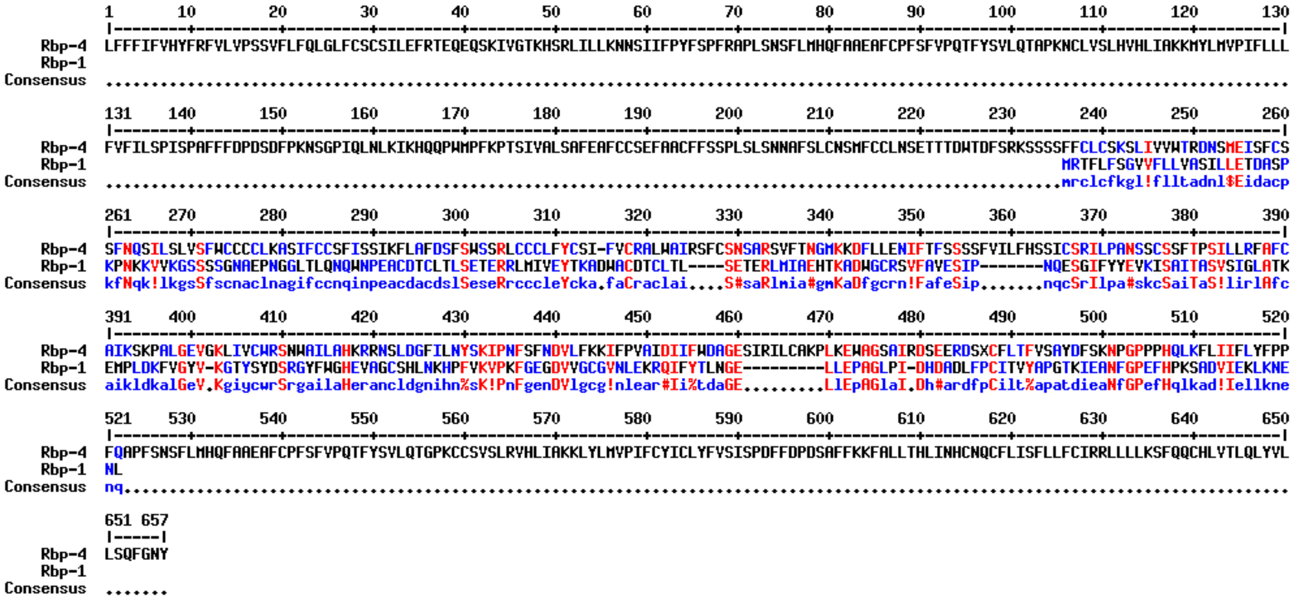

Fig. S4. Protein sequence similarity between Rbp-4 and Rbp-1 (ACJ14490.1 RBP1 [*Globodera pallida*]

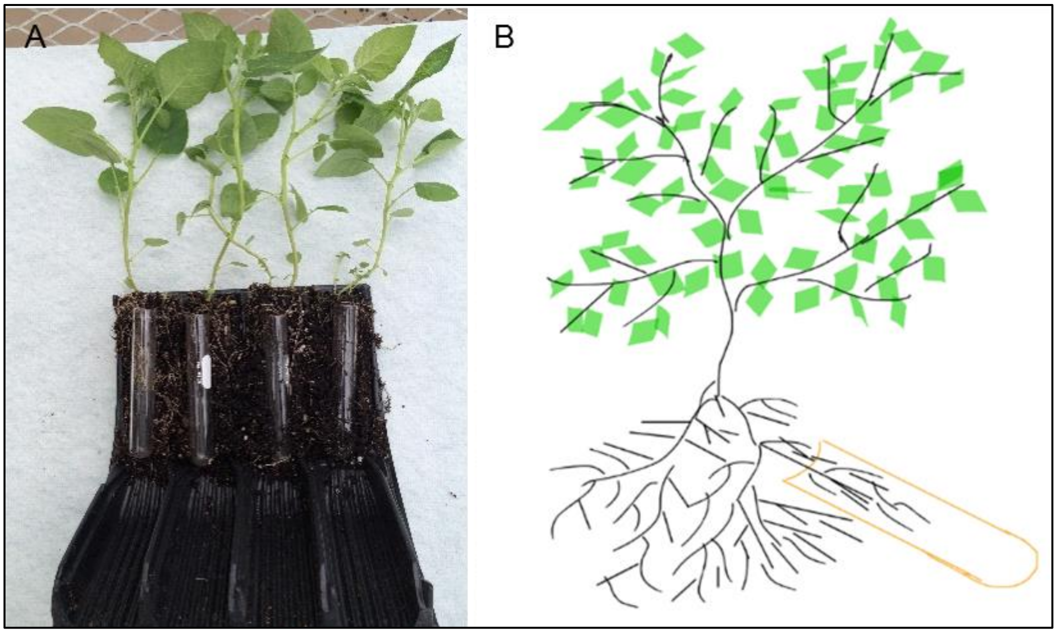

Fig. S5. Single root inoculation technique used for *Globodera pallida* infection. A, *Solanum tuberosum* growing in root trainers, a single root was transferred to the glass tube prior to inoculating with *G. pallida*; B, Prototype of single root inoculation used in the present study.

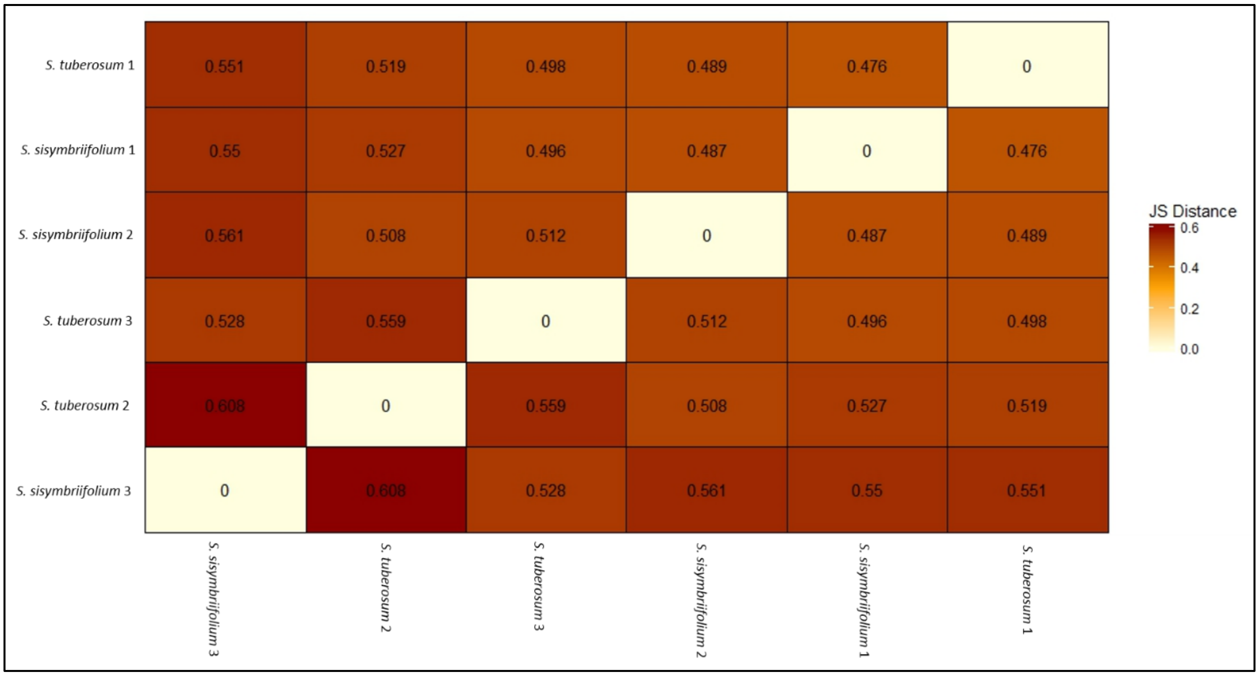

Fig. S6. Distance matrix showing insight into the relationship between the samples from susceptible *Solanum tuberosum* and resistant *Solanum sisymbriifolium* with three biological replicates*.* The color scale given on the right represents the distance between different samples.
